# GenHap: a novel computational method based on genetic algorithms for haplotype assembly

**DOI:** 10.1186/s12859-019-2691-y

**Published:** 2019-04-18

**Authors:** Andrea Tangherloni, Simone Spolaor, Leonardo Rundo, Marco S. Nobile, Paolo Cazzaniga, Giancarlo Mauri, Pietro Liò, Ivan Merelli, Daniela Besozzi

**Affiliations:** 1 Department of Informatics, Systems and Communication (DISCo), University of Milano-Bicocca, Viale Sarca 336, U14 Building, Milan, 20126 Italy; 2 Department of Human and Social Sciences, University of Bergamo, Piazzale Sant’Agostino 2, Bergamo, 24129 Italy; 3 Computer Laboratory, University of Cambridge, 15 JJ Thomson Avenue, Cambridge, CB3 0FD UK; 4 Institute of Biomedical Technologies, Italian National Research Council, Via Fratelli Cervi 93, Segrate (MI), 20090 Italy; 5 Institute of Molecular Bioimaging and Physiology, Italian National Research Council, Contrada Pietrapollastra-Pisciotto, Cefalù (PA), 90015 Italy; 6 SYSBIO.IT Centre of Systems Biology, Piazza della Scienza 2, Milan, 20126 Italy

**Keywords:** Haplotype assembly, Future-generation sequencing, Genetic algorithms, Combinatorial optimization, Weighted minimum error correction problem

## Abstract

**Background:**

In order to fully characterize the genome of an individual, the reconstruction of the two distinct copies of each chromosome, called haplotypes, is essential. The computational problem of inferring the full haplotype of a cell starting from read sequencing data is known as haplotype assembly, and consists in assigning all heterozygous Single Nucleotide Polymorphisms (SNPs) to exactly one of the two chromosomes. Indeed, the knowledge of complete haplotypes is generally more informative than analyzing single SNPs and plays a fundamental role in many medical applications.

**Results:**

To reconstruct the two haplotypes, we addressed the weighted Minimum Error Correction (wMEC) problem, which is a successful approach for haplotype assembly. This NP-hard problem consists in computing the two haplotypes that partition the sequencing reads into two disjoint sub-sets, with the least number of corrections to the SNP values. To this aim, we propose here GenHap, a novel computational method for haplotype assembly based on Genetic Algorithms, yielding optimal solutions by means of a global search process. In order to evaluate the effectiveness of our approach, we run GenHap on two synthetic (yet realistic) datasets, based on the Roche/454 and PacBio RS II sequencing technologies. We compared the performance of GenHap against HapCol, an efficient state-of-the-art algorithm for haplotype phasing. Our results show that GenHap always obtains high accuracy solutions (in terms of haplotype error rate), and is up to 4× faster than HapCol in the case of Roche/454 instances and up to 20× faster when compared on the PacBio RS II dataset. Finally, we assessed the performance of GenHap on two different real datasets.

**Conclusions:**

Future-generation sequencing technologies, producing longer reads with higher coverage, can highly benefit from GenHap, thanks to its capability of efficiently solving large instances of the haplotype assembly problem. Moreover, the optimization approach proposed in GenHap can be extended to the study of allele-specific genomic features, such as expression, methylation and chromatin conformation, by exploiting multi-objective optimization techniques. The source code and the full documentation are available at the following GitHub repository: https://github.com/andrea-tango/GenHap.

## Background

Somatic human cells are diploids, that is, they contain 22 pairs of homologous chromosomes and a pair of sex chromosomes, one copy inherited from each parent. In order to fully characterize the genome of an individual, the reconstruction of the two distinct copies of each chromosome, called haplotypes, is essential [1]. The process of inferring the full haplotype information related to a cell is known as haplotyping, which consists in assigning all heterozygous Single Nucleotide Polymorphisms (SNPs) to exactly one of the two chromosome copies. SNPs are one of the most studied genetic variations, since they play a fundamental role in many medical applications, such as drug-design or disease susceptibility studies, as well as in characterizing the effects of SNPs on the expression of phenotypic traits [2]. This information can be valuable in several contexts, including linkage analysis, association studies, population genetics and clinical genetics [3]. Obviously, the complete set of SNPs of an individual (i.e., his/her haplotypes) is generally more informative than analyzing single SNPs, especially in the study of complex disease susceptibility.

Since a direct experimental reconstruction of haplotypes still requires huge sequencing efforts and is not cost-effective [4], computational approaches are extensively used to solve this problem. In particular, two classes of methods exist for haplotype phasing [3]. The first class consists of statistical methods that try to infer haplotypes from genotypes sampled in a population. These data, combined with datasets describing the frequency by which the SNPs are usually correlated in different populations, can be used to reconstruct the haplotypes of an individual. The second class of methods directly leverages sequencing data: in this case, the main goal is to partition the entire set of reads into two sub-sets, exploiting the partial overlap among them in order to ultimately reconstruct the corresponding two different haplotypes of a diploid organism [5]. The effectiveness of these methods was limited by the length of the reads produced by second-generation sequencing technologies, which might be not long enough to span over a relevant number of SNP positions. This results in the reconstruction of short haplotype blocks [6, 7], since reads do not cover adjacent SNP positions adequately, hindering the possibility of reconstructing the full haplotypes. However, in recent years the development of new sequencing technologies paved the way to the advent of the third-generation of sequencing platforms, namely PacBio RS II (Pacific Biosciences of California Inc., Menlo Park, CA, USA) [8, 9] and Oxford Nanopore MinION (Oxford Nanopore Ltd., Oxford, United Kingdom) [10], which are able to produce reads covering several hundreds of kilobases and spanning different SNP loci at once. Unfortunately, the increased length comes at the cost of a decreased accuracy with respect to short and precise second-generation sequencing technologies, like NovaSeq (Illumina Inc., San Diego, CA, USA) [11]; thus, in order to obtain reliable data, the read coverage should be increased.

Among the computational methods for haplotype assembly, the Minimum Error Correction (MEC) is one of the most successful approaches. This problem consists in computing the two haplotypes that partition the sequencing reads into two disjoint sets with the least number of corrections to the SNP values [12]. Unfortunately, MEC was proven to be NP-hard [13]. A weighted variant of MEC, named weighted MEC (wMEC), was then proposed in [14]: the weights represent the confidence for the presence of a sequencing error, while the correction process takes into account the weight associated with each SNP value of a read. These error schemes generally regard phred-scaled error probabilities and are very valuable for processing long reads generated by third-generation sequencing technologies, as they are prone to high sequencing error rates [5].

Several assembly approaches have been already proposed in literature. Due to the NP-hardness of the MEC problem, some methods exploit heuristic strategies. Two noteworthy approaches are ReFHap [15], which is based on a heuristic algorithm for the Max-Cut problem on graphs, and ProbHap [16], which generalizes the MEC formulation by means of a probabilistic framework. In [12], Wang et al. proposed a meta-heuristic approach based on Genetic Algorithms (GAs) to address an extended version of the MEC problem, called MEC with Genotype Information (MEC/GI), which also considers genotyping data during the SNP correction process. A similar work was presented in [17], where GAs are used to solve the MEC problem by using a fitness function based on a majority rule that takes into account the allele frequencies. The results shown in [17] are limited to a coverage up to 10× and a haplotype length equal to 700. More recently, an evolutionary approach called Probabilistic Evolutionary Algorithm with Toggling for Haplotyping (PEATH) was proposed in [18]. PEATH is based on the Estimation of Distribution Algorithm (EDA), which uses the promising individuals to build probabilistic models that are sampled to explore the search space. This meta-heuristic deals with noisy sequencing reads, reconstructing the haplotypes under the all-heterozygous assumption. These algorithms present some limitations, as in the case of ReFHap [15], ProbHap [16] and PEATH [18], which assume that the columns in the input matrix correspond to heterozygous sites [19]. However, this all-heterozygous assumption might be incorrect for some columns, and these algorithms can only deal with limited reads coverages. For example, ProbHap [16] can handle long reads coverage values up to 20×, which is not appropriate for higher coverage short-read datasets; on the other hand, it works better with very long reads at a relatively shallow coverage (≤12×).

More recently, a tool based on a dynamic programming approach, called WhatsHap, was presented [5]. WhatsHap is based on a fixed parameter tractable algorithm [20, 21], and leverages the long-range information of long reads; however, it can deal only with datasets of limited coverage up to ∼20×. A parallel version of WhatsHap has been recently proposed in [22], showing the capability to deal with higher coverages up to ∼25×. An alternative approach, called HapCol [23], uses the uniform distribution of sequencing errors characterizing long reads. In particular, HapCol exploits a new formulation of the wMEC problem, where the maximum number of corrections is bounded in every column and is computed from the expected error rate. HapCol can only deal with instances of relatively small coverages up to ∼25−30×.

To sum up, even though high-throughput DNA sequencing technologies are paving the way for valuable advances in clinical practice, analyzing such an amount of data still represents a challenging task. This applies especially to clinical settings, where accuracy and time constraints are critical [24].

In order to tackle the computational complexity of the haplotyping problem, in this work we propose GenHap, a novel computational method for haplotype assembly based on Genetic Algorithms (GAs). GenHap can efficiently solve large instances of the wMEC problem, yielding optimal solutions by means of a global search process, without any a priori hypothesis about the sequencing error distribution in reads. The computational complexity of the problem is overcome by relying on a *divide-et-impera* approach, which provides faster and more accurate solutions compared with the state-of-the-art haplotyping tools.

The paper is structured as follows. In the next section, we briefly introduce the haplotyping problem, and describe in detail the GenHap methodology along with its implementation. Then, we show the computational performance of GenHap, extensively comparing it against HapCol. We finally provide some conclusive remarks and future improvements of this work.

## Methods

### Problem formulation

Given *n* positions on two homologous sequences belonging to a diploid organism and *m* reads obtained after a sequencing experiment, we can reduce each read to a fragment vector **f**∈{0,1,−}^*n*^, where 0 denotes a position that is equal to the reference sequence, 1 denotes a SNP with respect to the reference sequence, and − indicates a position that is not covered by the read. We define a haplotype as a vector **h**∈{0,1}^*n*^, that is, the combination of SNPs and wild-type positions belonging to one of the two chromosomes. Given the two haplotypes **h**_1_ and **h**_2_—which refer to the first and second copy of the chromosome, respectively—a position *j* (with *j*∈{1,…,*n*}) is said to be heterozygous if and only if $h_{1_{j}} \neq h_{2_{j}}$, otherwise *j* is homozygous.

**Fig. 1:**
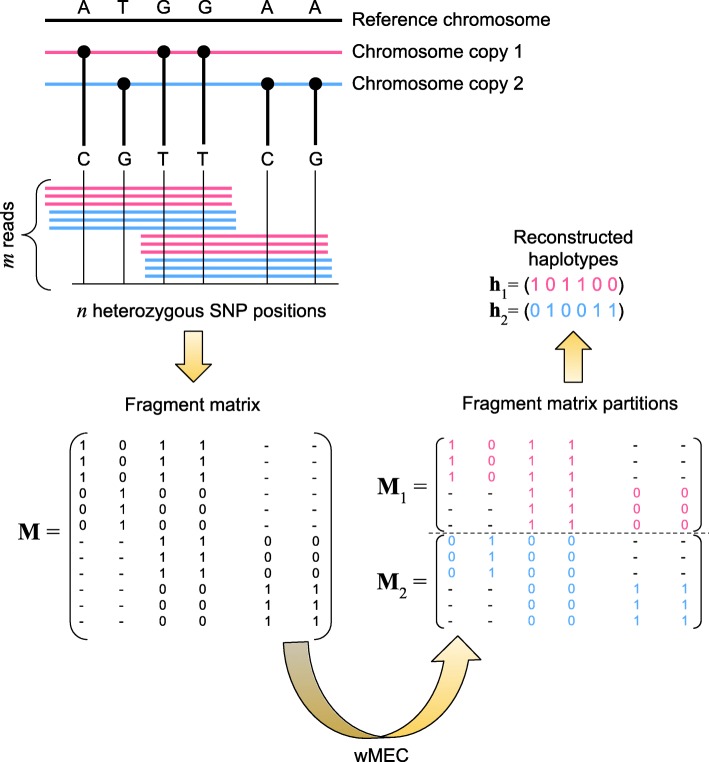
Simplified workflow of the haplotype assembly process. Raw sequencing data are initially aligned, defining *m* reads. Every position of the two chromosome copies is compared against a reference chromosome. The black solid points denote *n* heterozygous positions, along with the corresponding nucleobases. The fragment matrix **M** is defined assigning 1 to SNP positions and 0 to wild-type positions. To reconstruct the two haplotypes **h**_1_ and **h**_2_ characterized by the least number of corrections to the SNP values among the 2^*n*^ candidate haplotypes, the wMEC problem is solved by partitioning the matrix **M** into two disjoint matrices **M**_1_ and **M**_2_

Let **M** be the “fragment matrix”, that is, the *m*×*n* matrix containing all fragments. Two distinct fragments **f** and **g** are said to be in conflict if there is a position *j* (with *j*∈{1,…,*n*}) such that *f*_*j*_≠*g*_*j*_ and *f*_*j*_,*g*_*j*_≠−, otherwise they are in agreement. **M** is conflict-free if there are two different haplotypes **h**_1_ and **h**_2_ such that each row *M*_*i*_ (with *i*∈{1,…,*m*}) is in agreement with either **h**_1_ or **h**_2_. The overall haplotype assembly process is outlined in Fig. 1.

We can extend the heterozygous and homozygous definition at the column level as follows: a column *c* of **M** is homozygous if all its values are either in {0,−} or in {1,−}, on the contrary *c* is heterozygous because its values are in {0,1,−}, meaning that both a SNP and a wild-type exist in that position. Finally, we can detect the case where two distinct fragments are in conflict, and measure their diversity by defining a distance *D*(·,·) that calculates the number of different values between two fragments. Namely, given **f**=(*M*_*i*1_,…,*M*_*in*_) and **g**=(*M*_*l*1_,…,*M*_*ln*_) of **M** (with *i*,*l*∈{1,…,*m*}), we consider:

1 $$  D(\mathbf{f}, \mathbf{g}) = \sum_{j=1}^{n} d(f_{j}, g_{j}),  $$

where *d*(*f*_*j*_,*g*_*j*_) is defined as:

2 $$  d(x,y) = \left\{\begin{array}{ll} 1, & \text{if}\ x \neq y, x \neq -, \text{ and}\ y \neq -\\ 0, & \text{otherwise} \end{array}\right..  $$

Equation (1) defines the *extended Hamming distance* between two ternary strings **f** and **g** [19], denoting the total number of positions wherein both characters of **f** and **g** belong to {0,1} but they are different according to Eq. (2).

If **M** is conflict-free, then it can be partitioned into two disjoint matrices **M**_1_ and **M**_2_, each one containing a set of conflict-free fragments. We can infer the two haplotypes **h**_1_ and **h**_2_ from **M**_1_ and **M**_2_, respectively, as follows:

3 $$ h_{k_{j}} = \left\{\begin{array}{ll} 1, & \text{if}\ N_{1_{j}}(\mathbf{M}_{k}) \geq N_{0_{j}}(\mathbf{M}_{k})\\ 0, & \text{otherwise} \end{array}\right.,  $$

where *j*∈{1,…,*n*}, *k*∈{1,2}, and $N_{0_{j}}(\mathbf {M}_{k})$, $N_{1_{j}}(\mathbf {M}_{k})$ denote the number of 0s and 1s in the *j*-th column, respectively. In such a way, **N**_0_(**M**_*k*_) is the vector consisting of the number of 0s of each column *j* using the reads of the partition **M**_*k*_, while **N**_1_(**M**_*k*_) is the vector consisting of the number of 1s of each column *j* represented by the partition **M**_*k*_.

In order to solve the wMEC problem, **N**_0_ and **N**_1_ are calculated using the *m*×*n* weight matrix **W**, representing the weight associated with each position in each fragment. As a matter of fact, **W** can be divided into the two disjoint partitions **W**_1_ and **W**_2_, whose row indices correspond to those in **M**_1_ and **M**_2_, respectively. We can extend Eq. (3) taking into account the weights as follows:

4 $$  h_{k_{j}} = \left\{\begin{array}{ll} 1, & \text{if}\ N_{1_{j}}(\mathbf{W}_{k}) \geq N_{0_{j}}(\mathbf{W}_{k})\\ 0, & \text{otherwise} \end{array}\right.,  $$

where *j*∈{1,…,*n*}, *k*∈{1,2}, and $N_{0_{j}}(\mathbf {W}_{k})$, $N_{1_{j}}(\mathbf {W}_{k})$ denote the sum of the weights associated with the 0 and 1 elements in the *j*-th column, respectively.

The distance *D*(·,·) given in Eq. (1) can be used also to evaluate the distance between a fragment and a haplotype, by means of the following error function:

5 $$  \mathcal{E}(\mathbf{M}_{1},\mathbf{M}_{2}, \mathbf{h}_{1}, \mathbf{h}_{2}) = \sum_{k=1}^{2} \sum_{\mathbf{f} \in \mathbf{M}_{k}} D(\mathbf{f}, \mathbf{h}_{k}).  $$

The best partitioning of **M** can be obtained by minimizing Eq. (5), inferring **h**_1_ and **h**_2_ with the least number of errors. Equation (5) is used as fitness function in GenHap.

### GenHap: haplotype assembly using GAs

GAs are population-based optimization strategies mimicking Darwinian processes [25–27]. In GAs, a population *P* of randomly generated individuals undergoes a selection mechanism and is iteratively modified by means of genetic operators (i.e., crossover and mutation). Among the existing meta-heuristics for global optimization, GAs are the most suitable technique in this context thanks to the discrete structure of the candidate solutions. This structure is well-suited to efficiently solve the intrinsic combinatorial nature of the haplotype assembly problem. In the most common formulation of GAs, each individual *C*_*p*_ (with *p*∈{1,…,|*P*|}) encodes a possible solution of the optimization problem as a fixed-length string of characters taken from a finite alphabet. Based on a quality measure (i.e., the fitness value), each individual is involved in a selection process in which individuals characterized by good fitness values have a higher probability to be selected for the next iteration. Finally, the selected individuals undergo crossover and mutation operators to possibly improve offspring and to introduce new genetic material in the population.

GenHap exploits a very simple and efficient structure for individuals, which encodes as a binary string a partition of the fragment matrix **M**. In particular, each individual $\phantom {\dot {i}\!}C_{p}=[C_{p_{1}}, C_{p_{2}}, \ldots, C_{p_{m}}]$ (with $\phantom {\dot {i}\!}p \in \{1, \ldots, |P|\}$) is encoded as a circular array of size *m* (i.e., the number of reads). In order to obtain the two partitions **M**_1_ and **M**_2_, *C*_*p*_ is evaluated as follows: if the *i*-th bit is equal to 0, then the read *i* belongs to **M**_1_; otherwise, the read *i* belongs to **M**_2_. Once the two partitions are computed, GenHap infers the haplotypes **h**_1_ and **h**_2_ by applying Eq. (4). Finally, Eq. (5) is exploited to calculate the number of errors made by partitioning **M** as encoded by each individual of *P*. This procedure is iterated until the maximum number of iterations *T* is reached, the number of errors is equal to 0 or the fitness value of the best individual does not improve for *θ*=⌈0.25·*T*⌉ iterations.

Among the different selection mechanisms employed by GAs (e.g., roulette wheel [25], ranking [26], tournament [27]), GenHap exploits the tournament selection to create an intermediate population *P*^′^, starting from *P*. In each tournament, *κ* individuals are randomly selected from *P* and the individual characterized by the best fitness value is added to *P*^′^. The size of the tournament *κ* is related to the selection pressure: if *κ* is large, then the individuals characterized by worse fitness values have a low probability to be selected, therefore the variability of *P*^′^ might decrease.

Afterwards, the genetic operators (i.e., crossover and mutation) are applied to the individuals belonging to *P*^′^ to obtain the offspring for the next iteration. GenHap exploits a single-point crossover with mixing ratio equal to 0.5. Crossover is applied with a given probability *c*_*r*_ and allows for the recombination of two parent individuals *C*_*y*_,*C*_*z*_∈*P*^′^ (for some $\phantom {\dot {i}\!}y, z \in \{1, \ldots, |P|\}$), generating two offspring that possibly have better characteristics with respect to their parents.

In order to increase the variability of the individuals, one or more elements of the offspring can be modified by applying the mutation operator. GenHap makes use of a classic mutation in which the elements $C_{p_{e}}$ (with *e*∈{1,…,*m*}) of the individual can be flipped (i.e., from 0 to 1 or vice-versa) with probability *m*_*r*_. Besides this mutation operator, GenHap implements an additional bit-flipping mutation in which a random number of consecutive elements of the individual is mutated according to probability *m*_*r*_. This operator is applied if the fitness value of the best individual does not improve for a given number of iterations (2 in our tests).

Finally, to prevent the quality of the best solution from decreasing during the optimization, GenHap exploits an elitism strategy, so that the best individual from the current population is copied into the next population without undergoing the genetic operators.

**Fig. 2:**
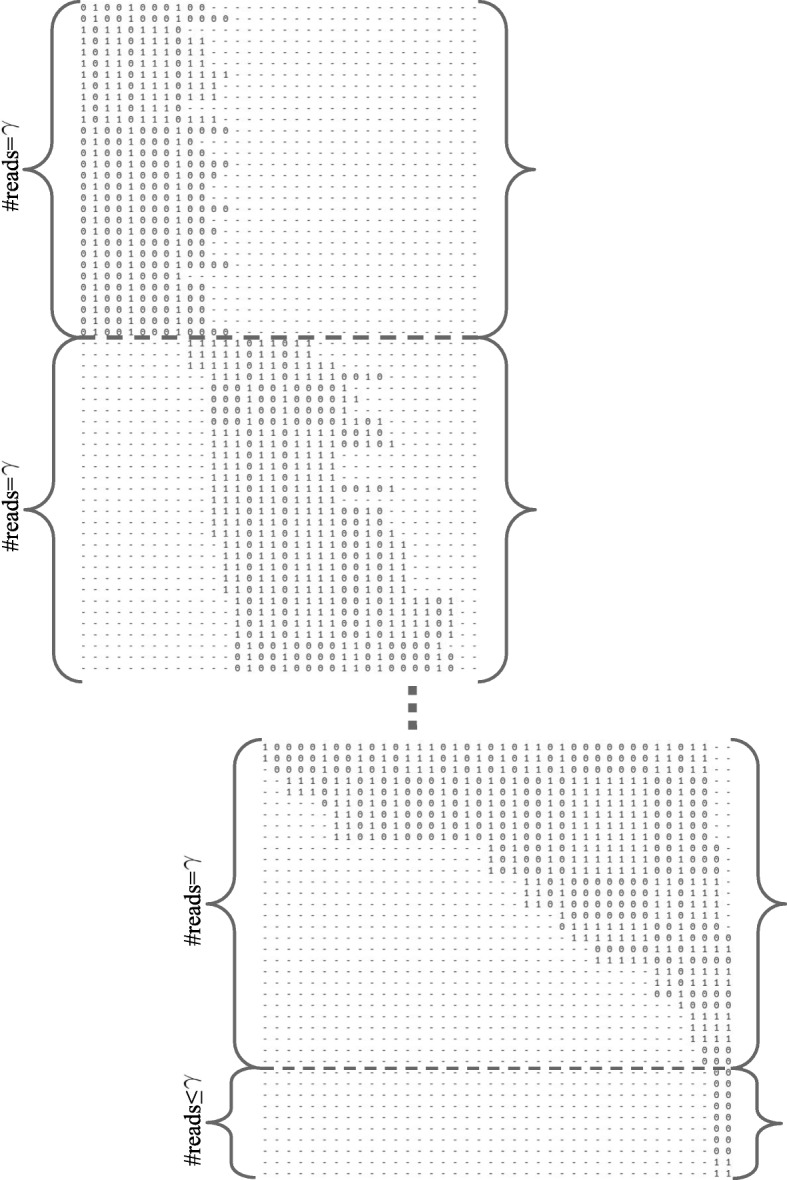
Scheme of the partition of the input matrix: the input matrix **M**∈{0,1,−}^*m*×*n*^ is split into sub-matrices consisting of *γ* reads, generating *Π*=⌊*m*/*γ*⌋ sub-problems that are solved independently by a GA instance. The last sub-matrix could have a number of reads lower than *γ*

Unlike the work in [12], GenHap solves the wMEC problem instead of the unweighted MEC formulation, by means of Eq. (4). Moreover, differently from the other heuristic strategies, such as ReFHap [15] and ProbHap [16], we did not assume the all-heterozygosity of the phased positions [19]. Under this assumption, every column corresponds to heterozygous sites, implying that **h**_1_ must be the complement of **h**_2_. In addition, since the required execution time as well as the problem difficulty increase with the number of reads and SNPs, to efficiently solve the wMEC problem we split the fragment matrix **M** into *Π*=⌊*m*/*γ*⌋ sub-matrices consisting of *γ* reads (see Fig. 2). Following a *divide-et-impera* approach [28], the computational complexity can be tackled by partitioning the entire problem into smaller and manageable sub-problems, each one solved by a GA that converges to a solution characterized by two sub-haplotypes with the least number of corrections to the SNP values. The solutions to the sub-problems achieved by the *Π* GA instances are finally combined. This approach is feasible thanks to the long reads with higher coverage produced by the second- and third-generation sequencing technologies. As a matter of fact, highly overlapping reads allow us to partition the problem into easier sub-problems, avoiding the possibility of obtaining incorrect reconstructions during the merging phase.

The parameter *γ*, used for the calculation of *Π*, depends on the coverage value and on the nature of the sequencing technology; its value must be set to avoid discrete haplotype blocks that do not exist in the input matrix **M**. Generally, the intervals where several independent historical recombination events occurred separate discrete blocks, revealing greater haplotype diversity for the regions spanning the blocks [7].

GenHap firstly detects all the haplotype blocks inside the fragment matrix **M** and then, in each block, it automatically sets *γ* equal to the mean coverage of that block to partition the reads. Notice that GenHap solves each block sequentially and independently, obtaining a number of haplotype pairs equal to the number of detected blocks. So doing, for each block GenHap proceeds by executing *Π* different GA optimizations, one for each sub-problem, calculating 2·*Π* sub-haplotypes. The length of the individuals is equal to *γ*, except for the last sub-problem that could have a number of reads smaller than *γ* (accordingly, the length of the individuals could be smaller than *γ*).

Since the problem is divided into *Π* sub-problems, two sub-problems referring to contiguous parts of the two chromosome copies might contain some overlapped positions that can be either homozygous or heterozygous. However, the reads covering an overlapped position might not be entirely included in the same sub-problem. For this reason, during the GA-based optimizations, all the phased positions are assumed to be heterozygous. If a position *j* is homozygous (i.e., all the reads covering this position have the same value, belonging to {0,−} or {1,−}, in both the sub-partitions and in every read covering it), then only one of the two sub-haplotypes will have the correct value. This specific value is correctly assigned to the sub-haplotype covered by the highest number of reads by following a majority rule. As soon as the two sub-haplotypes are obtained, all the possible uncorrected heterozygous sites are removed and the correct homozygous values are assigned by checking the columns of the two sub-partitions. Finally, once all sub-problems in *Π* are solved, GenHap recombines the sub-haplotypes to obtain the two entire haplotypes **h**_1_ and **h**_2_ of the block under analysis.

GenHap is also able to find and mask the ambiguous positions by replacing the 0 or 1 value with a *X* symbol. We highlight that an ambiguous position is a position covered only by the reads belonging to one of the two haplotypes.

### Implementation

**Fig. 3:**
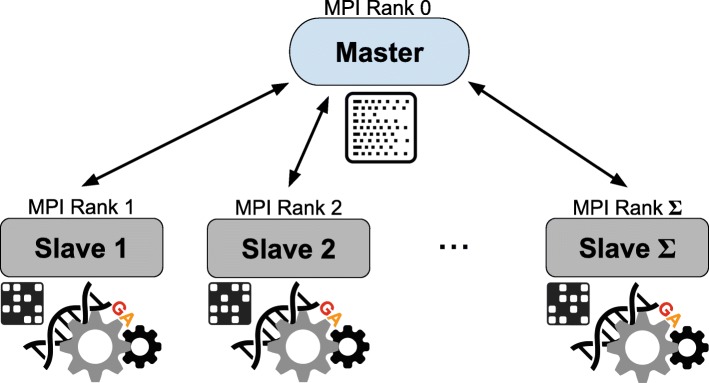
Scheme of the Master-Slave implementation of GenHap: the Master process orchestrates all the *Σ* Slaves sending one or more sub-partitions to each Slave, which then solves the assigned wMEC sub-task

In order to efficiently solve the wMEC problem and tackle its computational complexity, GenHap detects the haplotype blocks inside the matrix **M** and then, for each block, it splits the portion of **M** into *Π* sub-matrices consisting of *γ* reads. So doing, the convergence speed of the GA is increased thanks to the lower number of reads to partition in each sub-problem with respect to the total number of reads of the whole problem. As shown in Fig. 3, the *Π* sub-matrices are processed in parallel by means of a *divide-et-impera* approach that exploits a Master-Slave distributed programming paradigm [29, 30] to speed up the overall execution of GenHap. This strategy allowed us to distribute the computation in presence of multiple cores. As a matter of fact, GenHap works by partitioning the initial set of reads into sub-sets and solving them by executing different GA instances. This strategy can be exploited in GenHap, as it solves the wMEC problem working on the rows of the fragment matrix **M**; on the contrary, HapCol works considering the columns of **M**, which cannot be independently processed in parallel.

The functioning of our Master-Slave implementation can be summarized as follows:

1. the Master allocates the resources and detects the haplotype blocks inside the fragment matrix. For each detected block, it partitions the portion of the matrix **M** into *Π* sub-matrices and offloads the data onto the available *Σ* Slaves (in real scenarios, *Σ*≪*Π*). During this phase, each Slave generates the initial population of the GA;
2. the *σ*-th Slave (with *σ*∈{1,…,*Σ*}) executes the assigned wMEC sub-task, running the GA for either *θ* non-improving iterations or *T* maximum iterations, independently of the other Slaves;
3. the process is iterated until all the wMEC sub-tasks are terminated;
4. the Master recombines the sub-solutions received from the Slaves, and returns the complete wMEC solution for the block under analysis.

GenHap was entirely developed using the C++ programming language exploiting the Message Passing Interface (MPI) specifications to leverage multi-core Central Processing Units (CPUs).

## Results

In this section we first describe the synthetic and real datasets used during the tests and present the results obtained to identify the best GA setting. Then, we discuss the performance achieved by GenHap with respect to HapCol [23], which was previously shown to be more efficient than the other existing methods for the haplotype assembly problem, both in terms of memory consumption and execution time.

### The analyzed datasets

In order to test the performance of GenHap, we generated two synthetic (yet realistic) datasets, each one consisting of instances obtained from a specific sequencing technology. In particular, we considered the Roche/454 genome sequencer (Roche AG, Basel, Switzerland), representing one of the next-generation sequencing (NGS) systems able to produce long and precise reads, and the PacBio RS II sequencer [9, 31], which is an emerging third-generation sequencing technology. Note that the reads produced by the Roche/454 sequencer are approximately 9-times shorter than those generated by the PacBio RS II system.

In order to generate the datasets, we exploited the General Error-Model based SIMulator (GemSIM) toolbox [32]. GemSIM is a software able to generate *in silico* realistic sequencing data. It relies on empirical error models and distributions learned from real NGS data, and simulates both single- and paired-end reads from a single genome, collection of genomes, or set of related haplotypes. GemSIM can in principle simulate data from any sequencing technology producing output data encoded in the FASTQ format [33], for raw reads, and Sequence Alignment/Map (SAM), for aligned reads. In this work, we exploited the error model for the Roche/454 sequencer, already available in GemSIM, and defined an additional error model for the PacBio RS II technology. The synthetic reads were generated from the reference sequence of the human chromosome 22 (UCSC Genome Browser, GRCh37/hg19 Feb. 2009 assembly [34]), in which random SNPs were inserted.

We exploited the GemHaps tool included in GemSIM [32] to generate a haplotype file starting from a given genome sequence, and specifying the number as well as the frequency of SNPs in each haplotype, denoted by *#*SNPs and *f*_SNPs_, respectively. Note that the SNP positions were randomly determined. Then, the resulting haplotype file was processed by GemReads, together with an error model file (generated by GemErr or supplied in GemSIM), a FASTA genome file (or directory), and the selected quality score offset. The resulting SAM file was converted into the compressed Binary Alignment/Map (BAM) format for a more efficient manipulation [35]. In order to store the SNPs, we exploited the Variant Call Format (VCF) [36], which is the most used format that combines DNA polymorphism data, insertions and deletions, as well as structural variants. Lastly, the BAM and VCF files were processed to produce a WhatsHap Input Format (WIF) file [5], which is the input of GenHap.

The two synthetic datasets are characterized by the following features: *i*) *#*SNPs∈{500,1000,5000,10000,20000} (equally distributed over the two haplotypes); *ii*) coverage cov∈{∼ 30×, ∼ 60×}; *iii*) average *f*_SNPs_∈{100,200}, which means one SNP every 100bp or 200bp [37, 38], varying the portion of genome onto which the reads were generated. Read lengths were set to 600bp and 5000bp for the Roche/454 and the PacBio RS II sequencers, respectively. The number of reads was automatically calculated according to the value of cov and the sequencing technologies, by means of the following relationship:

6 $$  \#\text{reads} = \text{cov} \cdot\frac{len(\text{genome})}{len(\text{read})},  $$

where *len*(genome) represents the length of the considered genome, which starts at a given position *x* and ends at position *y*=*x*+*f*_SNPs_·*#*SNPs.

In order to test the performance of GenHap on real sequencing data, we exploited a WIF input file present in [39], which was generated starting from high-quality SNP calls and sequencing data made publicly available by the Genome in a Bottle (GIAB) Consortium [40]. In particular, we exploited data produced by the PacBio technology and limited to the chromosome 22 of the individual NA12878. Moreover, we tested GenHap on an additional real dataset available at [41]. As for the previous dataset, we limited our analysis to chromosome 22. The available BAM file–containing long reads with high-coverage produced with the PacBio RS II sequencing technology–and the VCF file were processed to obtain a WIF input file as described above.

### GA setting analysis

As a first step, the performance of GenHap was evaluated to determine the best settings for the haplotype assembly problem. We considered different instances for two sequencing technologies employed (i.e., Roche/454 and PacBio RS II), and we varied the settings of GenHap used throughout the optimization process, as follows:

- size of the population |*P*|∈{50,100,150,200};
- crossover rate *c*_*r*_∈{0.8,0.85,0.9,0.95};
- mutation rate *m*_*r*_∈{0.01,0.05,0.1,0.15}.

In all tests, the size of the tournament is fixed to *κ*=0.1·|*P*| and the maximum number of iterations is *T*=100. A total of 6 different instances (3 resembling the Roche/454 sequencer and 3 the PacBio RS II sequencer) were generated by considering *#*SNPs∈{500,1000,5000} and *f*_SNPs_=100.

We varied one setting at a time, leading to 64 different settings tested and a total number of 64×6=384 GenHap executions. These tests highlighted that, for each value of |*P*|, the best settings are:

1. |*P*|=50, *p*_*c*_=0.9, *p*_*m*_=0.05;
2. |*P*|=100, *p*_*c*_=0.9, *p*_*m*_=0.05;
3. |*P*|=150, *p*_*c*_=0.95, *p*_*m*_=0.05;
4. |*P*|=200, *p*_*c*_=0.95, *p*_*m*_=0.05.

**Fig. 4:**
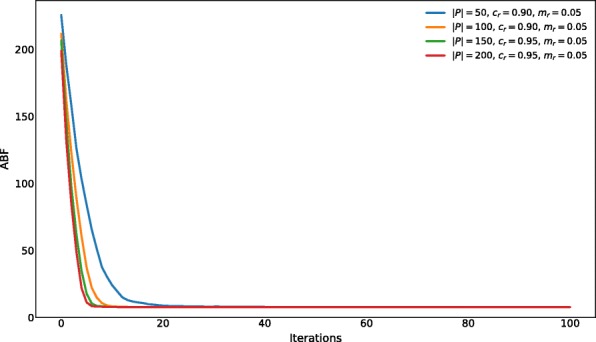
Comparison of the ABF achieved by GenHap with the best parameterizations found for each value of |*P*| tested here. The ABF was computed over the results of the optimization of instances characterized by *#*SNPs∈{500,1000,5000} and *f*_SNPs_=100

Figure 4 shows the comparison of the performance achieved by GenHap with the settings listed above, where the Average Best Fitness (ABF) was computed by taking into account, at each iteration, the fitness value of the best individuals over the 6 optimization processes. Even though all settings allowed GenHap to achieve almost the same final ABF value, we observe that the convergence speed increases with the size of the population. On the other hand, also the running time of GenHap increases with the size of the population. In particular, the executions lasted on average 1.41 s, 2.33 s, 3.52 s, 4.95 s with |*P*|∈{50,100,150,200}, respectively, running on one node of the Advanced Computing Center for Research and Education (ACCRE) at Vanderbilt University, Nashville, TN, USA. The node is equipped with 2 Intel^®^ Xeon^®^ E5-2630 v3 (8 cores at 2.40 GHz) CPUs, 240 GB of RAM and CentOS 7.0 operating system. To perform the tests we exploited all 8 physical cores of a single CPU.

Considering these preliminary results, we selected the parameter settings |*P*|=100, *c*_*r*_=0.9, *m*_*r*_=0.05, as the best trade-off between convergence speed (in terms of ABF) and running time.

### Performance of GenHap

The performance achieved by GenHap was compared with those obtained by HapCol [23], which was shown to outperform the main available haplotyping approaches. In particular, we exploited here a more recent version of HapCol, capable of dealing with haplotype blocks [39]. The same computational platform used for the setting analysis of GenHap was used to execute all the tests on the two synthetic datasets described above.

We stress the fact that GenHap was compared against HapCol only on the instances with cov≃30×, since HapCol is not capable of solving instances with higher coverage values (i.e., the algorithm execution halts when a column covered by more than 30 reads is found).

Considering the two sequencing technologies, we generated 15 different instances for each value of *#*SNPs and *f*_SNPs_. The performance was then evaluated by computing (*i*) the average haplotype error rate (*HE*), which represents the percentage of SNPs erroneously assigned with respect to the ground truth [42], and (*ii*) the average running time.

**Fig. 5:**
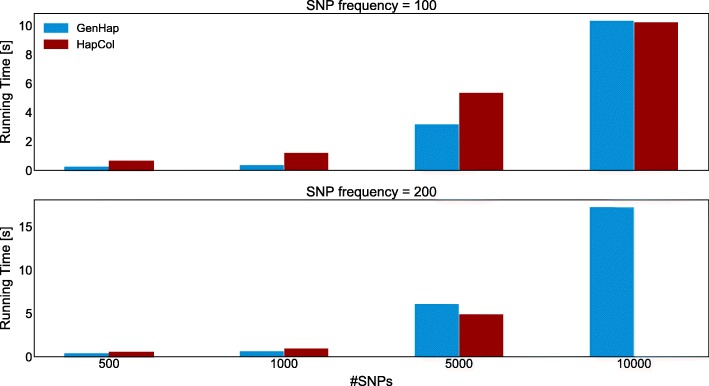
Comparison of the average running time required by GenHap (blue bars) and HapCol (red bars) computed over 15 instances for each value of *#*SNPs∈{500,1000,5000} obtained with the Roche/454 sequencing technology, cov≃30× and *f*_SNPs_=100. In the case of *f*_SNPs_=200 and *#*SNPs=10000, HapCol was not able to complete the execution on all the 15 instances

As shown in Table 1, in the instances generated using the Roche/454 sequencing technology with *f*_SNPs_=100, both GenHap and HapCol reconstructed the two haplotypes, achieving an average *HE* lower than 0.2*%* with a negligible standard deviation in the case of *#*SNPs∈{500,1000,5000}. GenHap inferred the haplotypes characterized by 10000 SNPs with an average *HE* lower than 2.5*%* and a standard deviation around 5%, while HapCol obtained an average *HE* equal to 6.55*%* with a standard deviation around 16%. For what concerns the running time, GenHap outperformed HapCol in all tests except in the case of *#*SNPs=10000, as shown in Fig. 5, being around 4× faster in reconstructing the haplotypes. In the case of *#*SNPs=10000, the running times are comparable, but GenHap obtains a lower *HE* than HapCol. In the instances generated using *f*_SNPs_=200 and *#*SNPs∈{500,1000}, both GenHap and HapCol reconstructed the two haplotypes, achieving an average *HE* lower than 0.1*%* with a negligible standard deviation. When *#*SNPs∈{5000,10000} are taken into account, GenHap inferred the haplotype pairs with an average *HE* lower than 3.65*%* and a standard deviation lower than 3.5*%*. Notice that HapCol was not able to complete the execution on all the 15 instances characterized by 10000 SNPs. As in the case of instances with *f*_SNPs_=100, GenHap is faster than HapCol in all tests, except in the case of *#*SNPs=5000.

**Table 1:** Comparison of GenHap and HapCol on the Roche/454 dataset with cov≃30×

			GenHap			HapCol		
*f*_SNPs_	cov	*#*SNPs	Avg *HE*	Std dev *HE*	Avg running time [s]	Avg *HE*	Std dev *HE*	Avg running time [s]
100	∼30×	500	0.04	0.08	0.21	0.00	0.00	0.62
		1000	0.09	0.08	0.36	0.00	0.00	1.20
		5000	0.18	0.06	3.17	0.01	0.03	5.35
		10000	2.50	5.52	10.33	6.55	16.38	10.23
200	∼30×	500	0.09	0.14	0.34	0.00	0.00	0.50
		1000	0.09	0.10	0.63	0.01	0.03	0.96
		5000	3.61	3.43	6.07	0.38	0.78	4.90
		10000	2.15	1.62	17.24	N/A	N/A	N/A

The performances were evaluated both in terms of *HE* and running time. The N/A symbol denotes that HapCol was not able to complete the execution on all the 15 instances

**Fig. 6:**
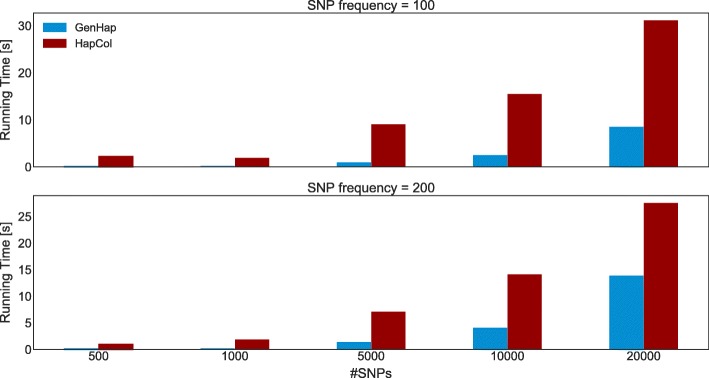
Comparison of the average running time required by GenHap (blue bars) and HapCol (red bars) computed over 15 instances for each *#*SNPs∈{500,1000,5000,10000,20000} obtained with the PacBio RS II sequencing technology, cov≃30×, *f*_SNPs_=100 (top) and *f*_SNPs_=200 (bottom)

For what concerns the PacBio RS II sequencing dataset, since this technology is characterized by a higher error rate with respect to the Roche/454 sequencer, both GenHap and HapCol reconstructed the two haplotypes with higher *HE* values (see Table 2). Nonetheless, the average *HE* value is lower than 2.5*%* with a standard deviation lower than 1% in all cases. Figure 6 shows the running time required by GenHap and HapCol to reconstruct the haplotypes. As in the case of the Roche/454 dataset, the running time increases with *#*SNPs, but GenHap always outperforms HapCol, achieving up to 20× speed-up.

**Table 2:** Comparison of GenHap and HapCol on the PacBio RS II dataset with cov≃30×

			GenHap			HapCol		
*f*_SNPs_	cov	*#*SNPs	Avg *HE*	Std dev *HE*	Avg running time [s]	Avg *HE*	Std dev *HE*	Avg running time [s]
100	∼30×	500	2.04	0.59	0.11	2.42	0.78	2.24
		1000	1.27	0.51	0.19	1.20	0.61	1.89
		5000	1.06	0.19	0.94	0.60	0.17	9.04
		10000	0.96	0.19	2.50	0.43	0.11	15.51
		20000	1.02	0.14	8.49	0.41	0.11	31.13
200	∼30×	500	2.09	0.52	0.14	1.73	0.42	0.95
		1000	1.70	0.24	0.22	1.09	0.41	1.84
		5000	1.05	0.18	1.39	0.54	0.11	7.10
		10000	1.13	0.18	4.09	0.51	0.17	14.13
		20000	1.02	0.13	13.86	0.33	0.05	27.55

The performances were evaluated both in terms of *HE* and running time

Table 3 lists the results obtained by GenHap on the instances of the Roche/454 dataset characterized by cov≃60×, *#*SNPs∈{500,1000,5000,10000} and *f*_SNPs_∈{100,200}. In all tests with *f*_SNPs_=100, GenHap was always able to infer the two haplotypes with high accuracy, indeed the average *HE* values are always lower than 0.15*%*. In the instances generated with *f*_SNPs_=200, GenHap reconstructed the haplotype pairs with an average *HE* lower than 0.2*%*. This interesting result shows that higher coverages can help during the reconstruction phase, allowing GenHap to infer more precise haplotypes.

**Table 3:** Results obtained by GenHap on the Roche/454 dataset with cov≃60×

			GenHap		
*f*_SNPs_	cov	*#*SNPs	Avg *HE*	Std dev *HE*	Avg running time [s]
100	∼60×	500	0.00	0.00	0.26
		1000	0.05	0.05	0.54
		5000	0.10	0.03	6.57
		10000	0.15	0.03	21.13
200	∼60×	500	0.00	0.00	0.37
		1000	0.07	0.09	0.89
		5000	1.13	1.72	11.17
		10000	2.00	1.02	53.77

The performances were evaluated both in terms of *HE* and running time

Regarding the PacBio RS II dataset, the achieved *HE* is on average lower than 1.25*%* with a standard deviation ≤0.4*%* (see Table 4). In particular, the average *HE* decreases when the value of *#*SNPs or the coverage increase, thus suggesting that higher cov values can considerably help in achieving a correct reconstruction of the two haplotypes. On the contrary, the running time increases at most linearly with respect to the coverage (see Table 4).

**Table 4:** Results obtained by GenHap on the PacBio RS II dataset with cov≃60×

			GenHap		
*f*_SNPs_	cov	*#*SNPs	Avg *HE*	Std dev *HE*	Avg running time [s]
100	∼60×	500	1.22	0.36	0.17
		1000	0.88	0.21	0.33
		5000	0.56	0.10	1.81
		10000	0.62	0.10	5.34
		20000	0.60	0.07	17.14
200	∼60×	500	1.22	0.37	0.22
		1000	0.79	0.27	0.36
		5000	0.53	0.09	3.26
		10000	0.45	0.08	8.01
		20000	0.49	0.05	27.15

The performances were evaluated both in terms of *HE* and running time

As a first test on real sequencing data, we exploited a WIF input file codifying the SNPs of the chromosome 22 generated from high-quality sequencing data made publicly available by the GIAB Consortium. This instance contains *#*SNPs≃27000 and *#*reads≃80000 with average and maximum coverages equal to 22 and 25, respectively. In [39], in order to down-sample the instances to the target maximum coverages of 30× allowed by HapCol, the authors applied a greedy-based pruning strategy. This procedure selects the reads characterized by high base-calling quality. GenHap detected and inferred the 305 different haplotype blocks in less than 10 min, obtaining approximately an 87% agreement with respect to the HapCol solution. This agreement was calculated considering every SNP of both haplotypes in each block.

We tested GenHap also on the chromosome 22 sequenced using the PacBio RS II technology (publicly available at [41]). This instance contains *#*SNPs≃28000 and *#*reads≃140000 with average and maximum coverages equal to 29 and 565, respectively. GenHap reconstructed the two haplotypes in about 10 min. This result shows that GenHap is capable of dealing with instances characterized by high coverages, avoiding pruning pre-processing steps.

## Discussion and conclusions

In this paper we presented GenHap, a novel computational method based on GAs to solve the haplotyping problem, which is one of the hot topics in Computational Biology and Bioinformatics. The performance of GenHap was evaluated by considering synthetic (yet realistic) read datasets resembling the outputs produced by the Roche/454 and PacBio RS II sequencers. The solutions yielded by GenHap are accurate, independently of the number, frequency and coverage of SNPs in the input instances, and without any a priori hypothesis about the sequencing error distribution in the reads.

In practice, our method was conceived to deal with data characterized by high-coverage and long reads, produced by recent sequencing techniques. The read accuracy achieved by novel sequencing technologies, such as PacBio RS II and Oxford Nanopore MinION, may be useful for several practical applications. In the case of SNP detection and haplotype phasing in human samples, besides read accuracy, a high-coverage is required to reduce possible errors due to few reads that convey conflicting information [43]. In [44], the authors argued that an average coverage higher than 30× is the *de facto* standard. As a matter of fact, the first human genome that was sequenced using Illumina short-read technology showed that, although almost all homozygous SNPs are detected at a 15× average coverage, an average depth of 33× is required to detect the same proportion of heterozygous SNPs.

GenHap was implemented with a distributed strategy that exploits a Master-Slave computing paradigm in order to speed up the required computations. We showed that GenHap is remarkably faster than HapCol [23], achieving approximately a 4× speed-up in the case of Roche/454 instances, and up to 20× speed-up in the case of the PacBio RS II dataset. In order to keep the running time constant when the number of SNPs increases, the number of available cores should increase proportionally with *#*SNPs.

Differently from the other state-of-the-art algorithms, GenHap was designed for taking into account datasets produced by the third-generation sequencing technologies, characterized by longer reads and higher coverages with respect to the previous generations. As a matter of fact, the experimental findings show that GenHap works better with the datasets produced by third-generation sequencers. Although several approaches have been proposed in literature to solve the haplotyping problem [5, 23], GenHap can be easily adapted to exploit Hi-C data characterized by very high-coverages (up to 90×), in combination with other sequencing methods for long-range haplotype phasing [45]. Moreover, GenHap can be also extended to compute haplotypes in organisms with different ploidity [46, 47]. Worthy of notice, GenHap could be easily reformulated to consider a multi-objective fitness function (e.g., by exploiting an approach similar to NSGA-III [48]). In this context, a possible future extension of this work would consist in introducing other objectives in the fitness function, such as the methylation patterns of the different chromosomes [49], or the gene proximity in maps achieved through Chromosome Conformation Capture (3C) experiments [50]. As a final note, we would like to point out that there is currently a paucity of up-to-date real benchmarks regarding the latest sequencing technologies. Therefore, collecting a reliable set of human genome sequencing data acquired with different technologies against the corresponding ground truth can be beneficial for the development of future methods.

## Abbreviations

3C: Chromosome Conformation Capture
ABF: Average Best Fitness
ACCRE: Advanced Computing Center for Research and Education
BAM: Binary Alignment/Map
CPU: Central Processing Unit
EDA: Estimation of Distribution Algorithm
GA: Genetic Algorithm
GeneSIM: General Error-Model based SIMulator
GIAB: Genome in a Bottle
HE: Haplotype Error rate
MEC: Minimum Correction Error
MPI: Message Passing Interface
NGS: Next-Generation Sequencing
PEATH: Probabilistic Evolutionary Algorithm with Toggling for Haplotyping
SAM: Sequence Alignment/Map
SNP: Single Nucleotide Polymorphism
VCF: Variant Call Format
WIF: WhatsHap Input Format
wMEC: Weighted Minimum Correction Error
